# Drug‐induced cardiomyopathy: Characterization of a rat model by [^18^F]FDG/PET and [^99m^Tc]MIBI/SPECT

**DOI:** 10.1002/ame2.12136

**Published:** 2020-10-10

**Authors:** Hailey Houson, Andria Hedrick, Vibhudutta Awasthi

**Affiliations:** ^1^ Research Imaging Facility Department of Pharmaceutical Sciences College of Pharmacy University of Oklahoma Health Science Center Oklahoma City OK USA; ^2^ Hexakit, Inc. Edmond OK USA

**Keywords:** [^18^F]‐fluorodeoxyglucose, [^99m^Tc]‐sestamibi, cardiomyopathy, imaging, myocardial infarction, positron emission tomography

## Abstract

**Background:**

Drug‐induced cardiomyopathy is a significant medical problem. Clinical diagnosis of myocardial injury is based on initial electrocardiogram, levels of circulating biomarkers, and perfusion imaging with single photon emission computed tomography (SPECT). Positron emission tomography (PET) is an alternative imaging modality that provides better resolution and sensitivity than SPECT, improves diagnostic accuracy, and allows therapeutic monitoring. The objective of this study was to assess the detection of drug‐induced cardiomyopathy by PET using 2‐deoxy‐2‐[^18^F]fluoro‐D‐glucose (FDG) and compare it with the conventional SPECT technique with [^99m^Tc]‐Sestamibi (MIBI).

**Methods:**

Cardiomyopathy was induced in Sprague Dawley rats using high‐dose isoproterenol. Nuclear [^18^F]FDG/PET and [^99m^Tc]MIBI/SPECT were performed before and after isoproterenol administration. [^18^F]FDG (0.1 mCi, 200‐400 µL) and [^99m^Tc]MIBI (2 mCi, 200‐600 µL) were administered via the tail vein and imaging was performed 1 hour postinjection. Isoproterenol‐induced injury was confirmed by the plasma level of cardiac troponin and triphenyltetrazolium chloride (TTC) staining.

**Results:**

Isoproterenol administration resulted in an increase in circulating cardiac troponin I and showed histologic damage in the myocardium. Visually, preisoproterenol and postisoproterenol images showed alterations in cardiac accumulation of [^18^F]FDG, but not of [^99m^Tc]MIBI. Image analysis revealed that myocardial uptake of [^18^F]FDG reduced by 60% after isoproterenol treatment, whereas that of [^99m^Tc]MIBI decreased by 45%.

**Conclusion:**

We conclude that [^18^F]FDG is a more sensitive radiotracer than [^99m^Tc]MIBI for imaging of drug‐induced cardiomyopathy. We theorize that isoproterenol‐induced cardiomyopathy impacts cellular metabolism more than perfusion, which results in more substantial changes in [^18^F]FDG uptake than in [^99m^Tc]MIBI accumulation in cardiac tissue.

## Graphical abstract

PET and SPECT nuclear images of cardiomyopathy.

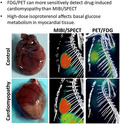

## 1 INTRODUCTION

MI is a leading cause of death in the United States and in most developed nations throughout the world.^1^ It can be a manifestation of acute mismatch in oxygen demand and supply to the cardiac tissue, or chronic heart failure attributed to left ventricular dysfunction. Several commonly used drugs including nonsteroidal anti‐inflammatory drugs, proton pump inhibitors, antidiabetics, antimigraines, Ca^2+^‐channel blockers, glucocorticoids, viral protease inhibitors, antineoplastics, and macrolide antibiotic classes are associated with increased risk of acute myocardial infarction or MI.^2^, ^3^ Drug‐induced myocardial dysfunction has led to the withdrawal of drugs from global or local markets, eg, Vioxx (rofecoxib), Bextra (valdecoxib), Zelnorm (tegaserod), and Reductil/Meridia (sibutramine).^4^, ^5^ Additionally, increased recreational drug use (cocaine, marijuana, etc) is beginning to expand drug‐induced MI (DIMI) as an acute event in otherwise normal young patients.^6^, ^7^, ^8^ It is suggested that most drug‐induced cardiotoxicity can be reversed if a prompt diagnosis is made.^9^, ^10^ Since DIMI can be associated with a much poorer prognosis than ischemic cardiac injury,^9^, ^11^ and since many drug users carry pre‐existing cardiac conditions, a need exists for a patient‐centered noninvasive technique to monitor DIMI and associated cardiovascular complications.

Although the clinical presentation of an individual with drug‐induced cardiomyopathy is similar to that of any patient reporting with angina or ischemia associated with atherosclerotic coronary artery disease (CAD), drug‐induced cardiomyopathy may occur in individuals who are not at risk for CAD. The classification and diagnosis of these patients is primarily based on chest pain, electrocardiogram (ECG) changes, and serum cardiac troponin levels. ECG can be inconclusive in many instances,^12^ whereas in many cases troponin levels may not accurately reflect cardiac status. For example, Suzuki et al found that samples collected ≤3 hours after symptom onset may by less sensitive.^13^ Cardiac troponin may take several hours to rise (12‐24 hours), so tests on admission may give misleading diagnosis. In addition, cardiac troponin remains elevated for up to 14 days of myocardial injury, which limits their usefulness in diagnosing reinfarction.^14^In order to increase the sensitivity and specificity of screening techniques for chemotherapy‐related cardiotoxicity, established guidelines also add functional assessment of left ventricular ejection fraction (LVEF) by Single Photon Emission Computed Tomography (SPECT) with ^99m^Tc‐based radiotracers.^10^, ^15^ Equilibrium radionuclide angiography is now considered the gold‐standard for chemotherapy‐related cardiotoxicity screening because it is more reproducible than electrocardiographic assessment of LVEF.^14^, ^16^, ^17^ However, as reported for adriamycin, resting LVEF alone may not be a sensitive indicator of drug‐induced cardiac changes.^10^, ^18^

Positron emission tomography (PET) can also be employed to determine myocardial perfusion, especially after the recent introduction of ^18^F‐labeled perfusion agents such as Flurpiridaz.^19^ In general, PET provides improved resolution, sensitivity of detection, less attenuation, and fewer imaging artifacts as compared to SPECT. We hypothesized that since metabolic changes are thought to precede morphologic changes and perfusion deficit in injured tissue, PET imaging with [^18^F]‐fluorodeoxyglucose (FDG) might provide a sensitive and early indicator of drug‐induced myocardial injury. PET using [^18^F]FDG as radiotracer is an established technique to determine metabolic correlates in cancer tissue.^20^ In this work, our objective was to evaluate the myocardial distribution of [^18^F]FDG and ^99m^Tc‐Sestamibi (MIBI) in cardiac injury induced by isoproterenol. [^99m^Tc]MIBI/SPECT is used in clinic to define perfusion of myocardial tissue.^21^ Isoproterenol is a sympathomimetic amine (β‐adrenergic agonist) which is used clinically as a bronchodilator in respiratory disorders and as a cardiac stimulant in cardiogenic shock. High‐dose administration of isoproterenol induces tachycardia, increases myocardial oxygen demand, produces reactive oxygen species, and results in widespread myocardial necrosis.^22^, ^23^ Its long‐term use is known to cause cardiac hypertrophy and fibrosis, modeling hypertrophic heart failure.^24^, ^25^ As such, isoproterenol‐treated rodents are commonly used as a standard model for MI.^22^, ^26^

## 2 METHODS

### 2.1 Materials

Isoproterenol was purchased from Sigma‐Aldrich (St. Louis, MO) and was used without further purification. Clinical grade [^18^F]FDG and [^99m^Tc]MIBI were purchased from the University of Oklahoma‐Nuclear Pharmacy (Oklahoma City, OK). Sprague Dawley rats were obtained from Envigo (formerly Harlan, Indianapolis, IN).

### 2.2 Rat model of isoproterenol‐induced cardiomyopathy

**FIGURE 1:**
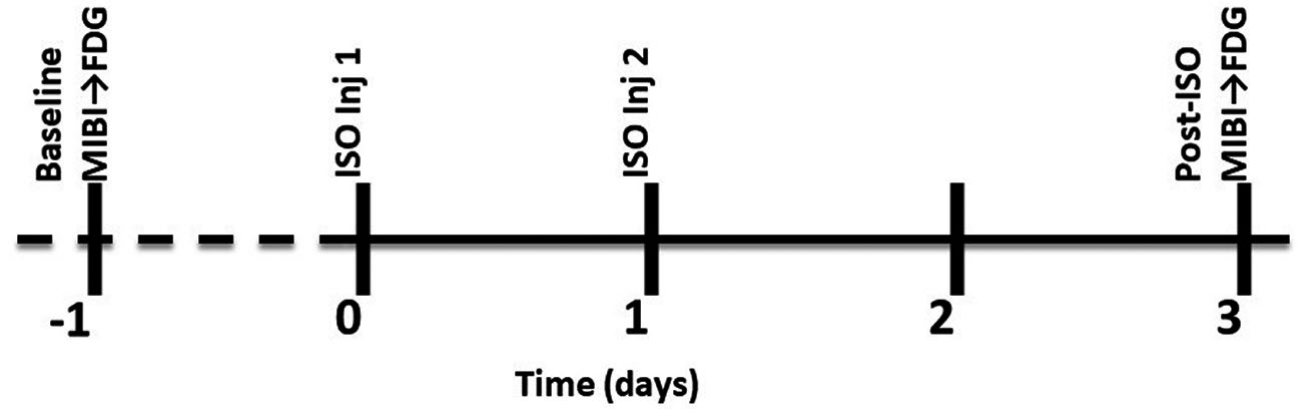
Timeline showing the schedule of procedures and image acquisition. Rats were imaged before and on the second day after isoproterenol (ISO) administration

All animal experiments were conducted according to a protocol approved by the Institutional Animal Care and Use Committee of the University of Oklahoma Health Sciences Center and in compliance with US Public Health Service's Policy on Humane Care and Use of Laboratory Animals, and Guide for the Care and Use of Laboratory Animals. To create a model of DIMI, we followed a previously reported method of isoproterenol‐induced cardiomyopathy.^22^ Male Sprague Dawley rats (250‐300 g) were housed in regular light/dark cycles and allowed to acclimatize for at least 5 days prior to experiments. Myocardial injury was induced by intraperitoneal administration of a sterile aqueous solution of isoproterenol (100 mg/kg) on two consecutive days. The timeline of PET and SPECT imaging sessions are described in Figure 1. A few of the rats recruited in this study were also subjected to another PET imaging session using ^18^F‐fluoroglucaric acid as has been reported in a separate article.^27^

### 2.3 Electrocardiography (ECG)

Lead 1 ECG of isoflurane‐anesthetized rats was recorded by a placing negative electrode on the right front paw, positive electrode on the left front paw, and ground lead on to the left hind paw. The electrodes were attached to the lead (CB Sciences C‐ISO‐255), bioamplifier (ETH‐225), and analog to digital converter (iWorx 118) and the signal was recorded using Labscribe 2.0 software (iWorx, Dover, NH). Before analysis of raw data, the signal was filtered to eliminate 60 Hz mains frequency.

### 2.4 Imaging

The sequence of treatment given to the rats is shown in Figure 1. To establish baseline images, rats were subjected to imaging with [^18^F]FDG/PET and [^99m^Tc]MIB/SPECT on the same day before isoproterenol administration. Imaging was repeated on the day after the second dose of isoproterenol (Figure 1). All radiotracer injections were given intravenously in the tail vein of anesthetized (2.5% isoflurane‐oxygen mixture) rats. Throughout the imaging period, anesthesia was maintained with a 2% isoflurane‐oxygen mixture. For SPECT, [^99m^Tc]MIBI (2 mCi, 0.5‐1 mL) was administered and imaging was performed 1 hour postinjection. Helical SPECT of the thoracic region in 24 frames of 60 seconds each was acquired on a NanoSPECT machine (Trifoil Imaging, Chatsworth, CA). For PET, [^18^F]FDG (0.1 mCi, 0.2‐0.4 mL) was injected and imaging was performed 1 hour postinjection on a PET‐CT dual modality machine (Gamma Medica Ideas, Northridge, CA). A fly‐mode CT of thoracic region was also acquired. After imaging, the rats were allowed to wake up and kept in their cage until the time of euthanasia.

SPECT acquisitions were reconstructed with HiSPECT reconstruction algorithm provided with the NanoSPECT system. The acquired list‐mode PET data were reconstructed by filtered back projection algorithm and fused with the CT to generate a composite PET‐CT image.

Standard uptake values (SUV) from the acquired PET and SPECT images were calculated by drawing a three‐dimensional volume of interest (VOI) around the heart using the CT image. Background was determined by placing a spherical VOI at the same level as the 4th sternebrae in the XY‐plane of the posterio‐medial region of the left lung. Background was subtracted from mean counts per voxel for the heart and the values were normalized to injected dose per gram bodyweight.

### 2.5 Cardiac troponin I assay

Blood samples were collected before euthanasia via the tail vein and plasma was separated by centrifugation (2430 *g* for 5 minutes). Cardiac Troponin I (cTnI) levels were determined in plasma by using a rat‐specific enzyme‐linked immunoassay kit obtained from Life Diagnostics (West Chester, PA). Plasma samples were diluted in 1:2 ratio with PBS before estimation.

### 2.6 Corticosterone enzyme‐linked immunosorbent assay (ELISA)

We measured the concentration of the stress hormone corticosterone in undiluted plasma by using an ELISA kit from Cayman Chemicals (Ann Arbor, Michigan). The results have been presented as percent of control values.

### 2.7 Evan's blue perfusion and 2,3,5‐Triphenyltetrazolium chloride (TTC) staining

After the final session of imaging and last blood sample collection, 1% percent Evan's blue was injected into the rats (2 mL/kg) before euthanasia. After 30 minutes, euthanasia was performed and their hearts were immediately collected. Whole hearts were pictured before freezing at −20°C for 2 hours. Afterwards, the hearts were sectioned into 2 mm slices and placed in 1% solution of TTC in PBS at 37°C with intermittent shaking. After 30 minutes of staining, the TTC solution was removed and replaced with 10% buffered formalin for overnight fixing. TTC stains viable tissue (red), but leaves dead/necrotic tissue unstained.

### 2.8 Data analysis

For statistical comparisons we employed GraphPad Prism 6 Software (GraphPad, La Jolla, CA). Two group comparisons were performed using an unpaired, two‐tailed Student's *T* Test. *P* value < .05 was the cut‐off for statistical significance. Data in figures are displayed as mean ± standard error of mean (SEM).

## 3 RESULTS

### 3.1 Isoproterenol‐induced myocardial injury

**FIGURE 2:**
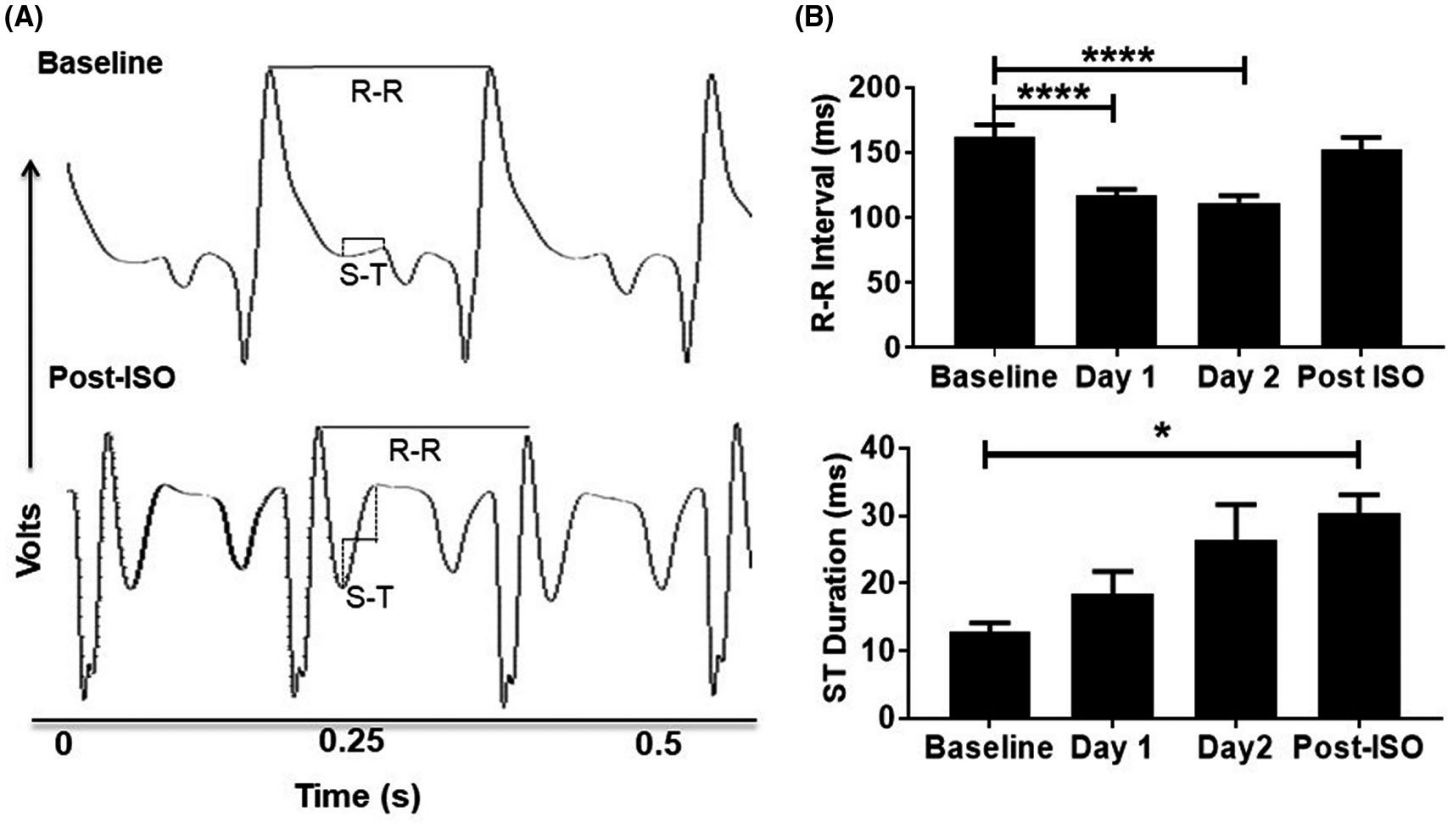
(A) A representative ECG recording showing qualitative changes in electrophysiology of rats before and after isoproterenol (ISO) administration. (B) R‐R interval decreased on the days of ISO treatment and returned to basal levels by day 3. ST segment duration remained increased through isoproterenol administration (^*^ *P* < .05, ^****^ *P* < .0001; n ≥ 6)

Figure 1 shows the timeline of imaging with respect to isoproterenol administrations. Isoproterenol‐treated rats showed qualitative changes in electrophysiology from Lead I recordings as compared to the control rats (Figure 2A). ECG changes occurred in all isoproterenol‐treated animals, however, because isoproterenol treatment creates nonspecific areas of necrosis, the changes were not consistent in magnitude across all isoproterenol‐treated rats. As expected, on the days of isoproterenol treatment the R‐R interval was significantly decreased indicating an increase in heart rate (Figure 2B, top panel). Over the course of isoproterenol treatment the ST duration increased (Figure 2B, bottom panel). These data indicate delayed ventricular repolarization which can be a result of prolonged action potentials in the purkinje fibers. The manifestation is known to occur with the administration of cardiotoxic agents.^28^

### 3.2 Cardiac troponin I and corticosterone in plasma

**FIGURE 3:**
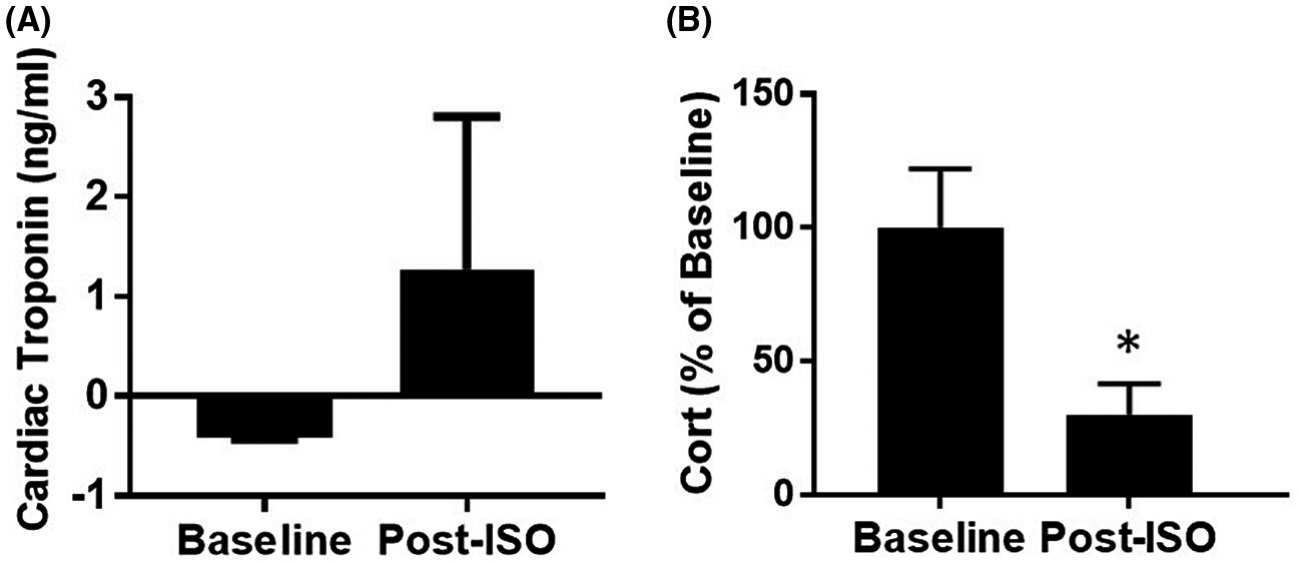
(A) Cardiac troponin (n = 6 baseline, n = 3 postisoproterenol) and (B) corticosterone levels (n = 5 baseline, n = 6 postisoproterenol). These biomarkers were determined in plasma collected at baseline and after isoproterenol (ISO) treatment (^*^ *P* < .05)

Circulating levels of cardiac troponin are highly sensitive clinical markers of acute myocardial pathology. Cardiac troponin was undetectable in control/baseline plasma (n = 6), and was increased in isoproterenol‐treated rat plasma (63 pg/mL, n = 4) (Figure 3A). While the troponin levels were increased, they were not as high as would be expected because the postisoproterenol plasma samples were obtained 24‐48 hours after isoproterenol administration. By this time, much of the circulating troponin is expected to be cleared from the blood.^29^ The plasma concentrations of corticosterone, a marker of physiologic stress, are shown (Figure 3B). Plasma levels of corticosterone in isoproterenol‐treated rats were significantly reduced as compared to the baseline levels as was expected with isoproterenol administration.^26^

### 3.3 Myocardial accumulation of [^99m^Tc]MIBI in cardiomyopathy

**FIGURE 4:**
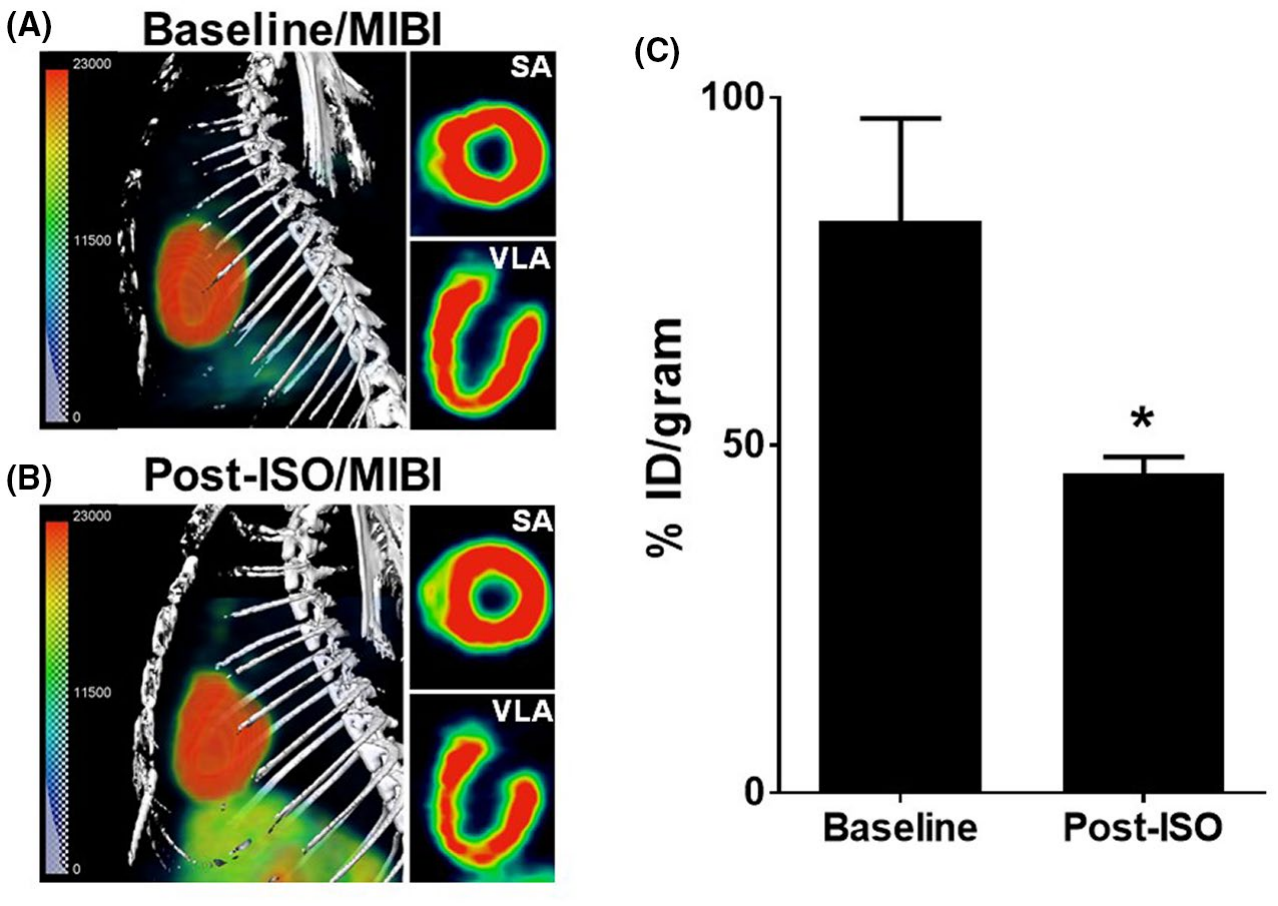
SPECT of isoproterenol (ISO)‐induced cardiomyopathy in rats using [^99m^Tc]MIBI. (A) Baseline SPECT images, (B) SPECT images after isoproterenol treatment, and (C) Myocardial SUV for [^99m^Tc]MIBI (^*^ *P* < .05, Students *T* test; n = 4 baseline and n = 5 postisoproterenol). Left lateral rendered images of the thoracic region are shown in left panels. Right‐hand panels depict short axis (SA) and vertical long axis (VLA) sections. The images were acquired 1 hour after [^99m^Tc]MIBI injection

Rats were imaged by a clinically utilized imaging agent, [^99m^Tc]MIBI, for myocardial perfusion. The left‐oblique composite views and tomograms of thoracic region at baseline and after isoproterenol treatments are shown in Figure 4A,B, respectively. In the composite view, it was difficult to find any difference in perfusion between baseline and postisoproterenol treatment images, but isoproterenol appeared to marginally reduce perfusion in the apex region as shown in vertical long axis (VLA) tomogram. When the images were analyzed by digital VOI analysis, we found a significant reduction in perfusion. [^99m^Tc]MIBI accumulation in isoproterenol‐treated hearts reduced by 45% as compared to the baseline values (from a mean value of 8.2 to 4.6; Figure 4C).

### 3.4 Myocardial [^18^F]FDG uptake in cardiomyopathy

**FIGURE 5:**
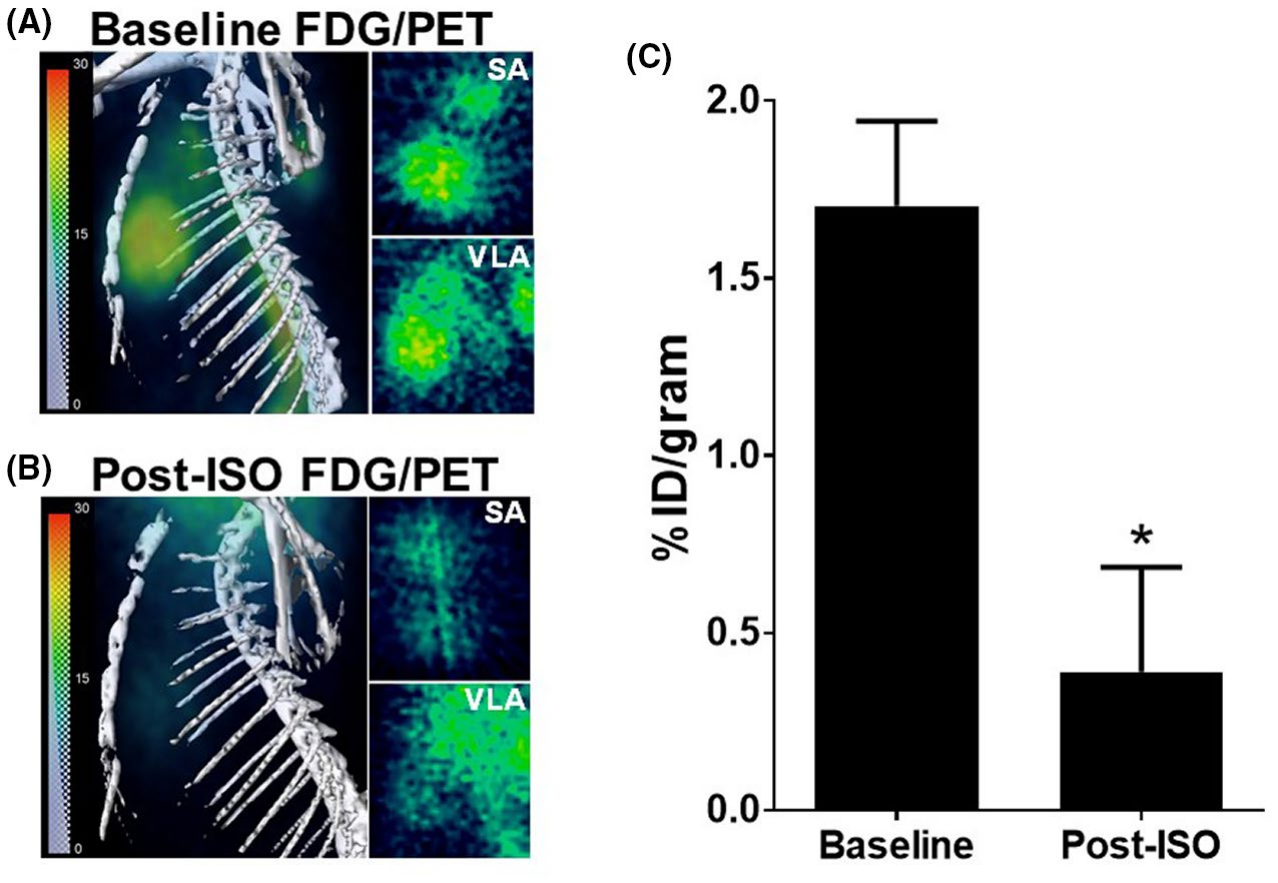
PET of isoproterenol (ISO)‐induced myopathy with [^18^F]FDG. (A) Baseline PET images, (B) PET images after isoproterenol treatment, and (C) Myocardial SUV for [^18^F]FDG (^*^ *P* < .05, Students *T* test; n = 7 baseline and n = 8 postisoproterenol). Like previous figure, left lateral rendered images of the thoracic region are shown. Right‐hand panels depict short axis (SA) and vertical long axis (VLA) sections. The images were acquired 1 hour after [^18^F]FDG injection

In order to investigate metabolic downregulation in isoproterenol‐induced myocardial injury, we employed PET using [^18^F]FDG as a an imaging marker. Figures 5A,B show the baseline images and postisoproterenol images of [^18^F]FDG uptake. The PET images were acquired 1 hour after injection of radiotracer. Just visual comparison of images in Figure 5A,B clearly showed that myocardial [^18^F]FDG uptake was significantly reduced in isoproterenol‐treated rats. Based on VOI‐based analysis of images, the reduction in FDG uptake was approximately 60% between the baseline value and postisoproterenol treatment value (from mean value of 3.2 to 1.3; Figure 5C).

### 3.5 Histology

**FIGURE 6:**
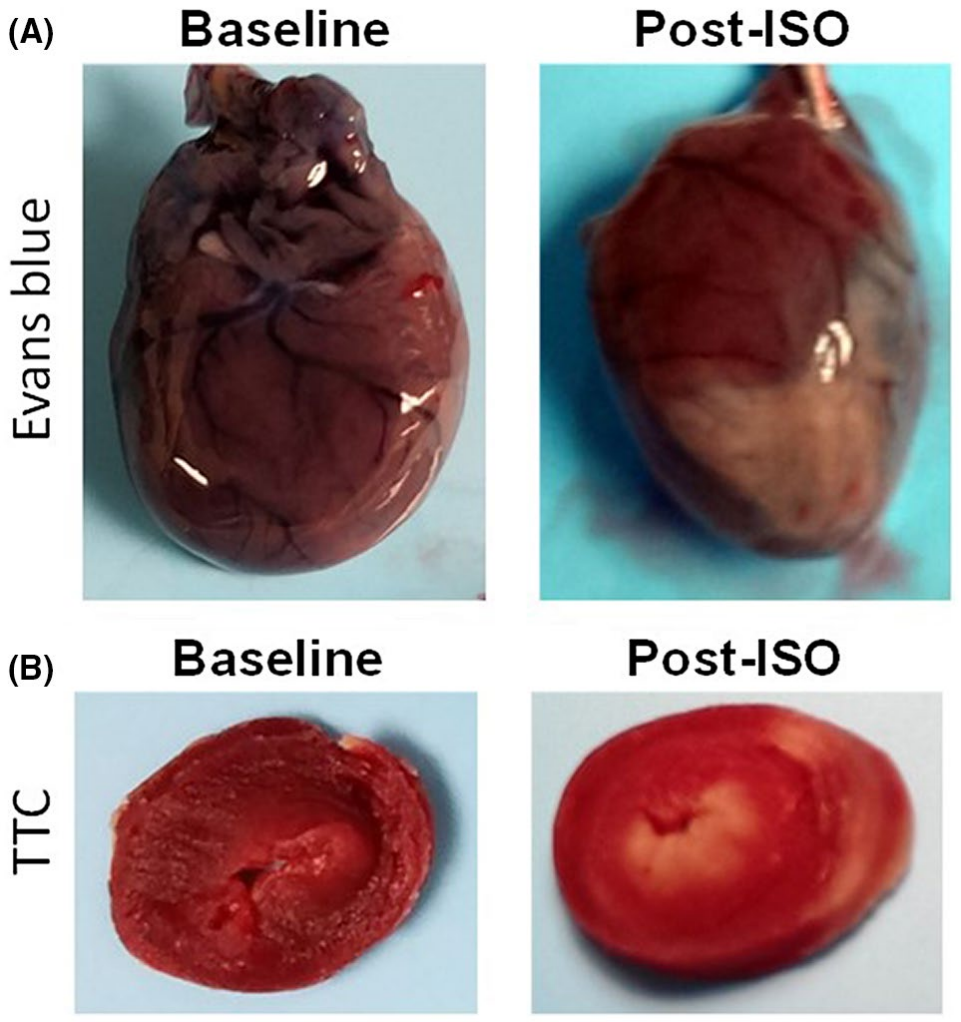
Gross histology of isoproterenol (ISO)‐induced cardiomyopathy in a rats. (A) Macroscopic histology of Evan's blue‐stained hearts where lack of perfusion is indicated by white areas. (B) TTC‐stained slices of heart, where dead tissue remains unstained (pale regions) and normal tissue is stained bright red

We used Evan's blue perfusion and TTC staining for assessment of myocardial injury at necropsy. After two consecutive days of isoproterenol injection, a representative whole heart perfused with 1% Evans blue showed that isoproterenol administration developed macroscopic regions of nonviable tissue and areas with poor perfusion (Figure 6A). Short‐axis sections of TTC‐stained heart slices (Figure 6B) showed areas of generalized and extensive necrosis (white regions) as a result of isoproterenol administration.

## 4 DISCUSSION

Acute MI is the most severe form of cardiac dysfunction which accounts for millions of deaths worldwide.^30^ Certain drugs can also induce acute coronary syndrome, however the exact incidence of acute MI caused by drugs or therapy in the general population is not known. Despite under‐reporting as well as selective‐reporting, chest pain and MI have been among the most common adverse events with serious outcomes reported to the FDA.^31^ In a long‐term follow‐up of survivors of pediatric malignancies who received chemotherapy, 23% had abnormal cardiac function and the incidence of cardiac dysfunction increased in a dose‐dependent manner.^32^ A more recent study reported that 7.8% of patients with a history of anthracycline use and/or radiation had abnormal ECG.^33^ Perhaps more concerning is the estimate that up to 50% of patients who develop left ventricular dysfunction after receiving an anthracycline and/or herceptin may not be on optimal treatment for abnormal cardiac condition.^9^

The biochemical etiology of DIMI is as varied as the drugs that cause it. Anthracycline anticancer drug doxorubicin is known to induce oxidative stress in myocardial tissue which results in lethal cardiomyopathy.^34^ Drugs, such as the anticancer multi‐kinase inhibitor sorafenib, have been reported to cause MI secondary to the induction of coronary artery spasm.^34^ Drugs of proteasome inhibitor class (bortezomib) may cause cardiomyopathy because of dysregulation of the ubiquitin‐proteasome system.^35^ Hodgkin's disease and breast cancer patients undergoing radiotherapy are at risk of cardiac damage because of myocardial exposure to high‐dose radiation.^31^ Many times, chronic use of drugs such as oral contraceptives, nicotine, rosiglitazone, cyclooxygenase‐2 inhibitors, and nonsteroidal anti‐inflammatory drugs may facilitate or increase the risk of coronary artery disease leading to myocardial ischemia and infarction.^31^

The mechanism for isoproterenol‐induced cardiomyopathy is based on the increase in myocardial oxygen demand caused by increased heart rate and myocardial contractility. In this respect, our rat model mimics clinical aspects of cardiomyopathy caused by drugs such cocaine and vasodilators (nifedipine and minoxidil). At the same time, isoproterenol has been known to increase oxidative stress in myocardial tissue.^18^, ^23^ Thus, it also represents toxicity caused by anthracyclines.^34^ As was evident from increased cTnI in plasma as well as altered ECG profile in our model, high‐dose isoproterenol treatment resulted in significant cardiac injury. Despite cTnI being elevated as compared to control, it was not as high as we expected, perhaps because the blood sampling was performed 48 hours past the last administration of isoproterenol. It has been shown by others that circulating cTnI levels are restored to near basal levels within 24 hours after isoproterenol administration.^29^ We also found a significant reduction in plasma corticosterone in isoproterenol‐treated rats. This was contrary to a general expectation that physiological stress increases blood levels of corticosterone. However, our findings corroborated the findings of Saroff and Wexler who reported increased clearance of corticosterone accompanied by its reduced serum protein‐binding in isoproterenol‐induced MI.^26^ Nevetheless, this observation is in contradiction to several clinical reports where plasma corticosterone was found to be elevated in MI patients.^36^, ^37^

Our imaging results indicate that [^18^F]FDG/PET is perhaps a better technique than perfusion imaging with [^99m^Tc]MIBI/SPECT. Thus, molecular imaging of glucose metabolism with PET may help to detect subclinical drug cardiotoxicity and complement traditional LVEF assessment. There have been very few clinical studies involving [^18^F]FDG/PET to evaluate drug‐induced cardiotoxicity.^16^ FDG is an analog of glucose in which the 2‐position hydroxyl group is replaced with radioactive fluorine‐18. It is taken up by cells in proportion to their glucose consumption and becomes trapped after the first step of glycolysis. Even though the primary source of energy relied upon by the heart is fatty acid oxidation, [^18^F]FDG is substantially taken up by normal heart tissue. In isoproterenol‐treated hearts [^18^F]FDG accumulation was reduced as compared to the control values, but it was still detectable in surrounding tissues including brown fat. These results are in accordance with the clinical and preclinical findings reported about [^18^F]FDG accumulation in congestive heart failure.^38^ We suspect that the decrease in cardiac [^18^F]FDG accumulation in isoproterenol‐treated rats is due to regions of nonviable tissue created by isoproterenol treatment. It is notable that smaller doses of isoproterenol have been shown to increase FDG uptake in brown fat and some tumor lines.^39^ Additionally, studies during doxorubicin treatment have shown increased uptake of FDG as treatment progressed.^40^ Perhaps, during early stages of myocardial perturbation caused by chemotherapeutic insult, glucose metabolism is enhanced as a reaction.

Unlike [^18^F]FDG, [^99m^Tc]MIBI is a cationic radiotracer with a significant ability to partition into the lipid phase. It accumulates in the myocardium in proportion to both the extent of myocardial perfusion and the viability of myocytes. It diffuses across the cell membrane and its intracellular trapping is dependent on mitochondrial membrane potentials present in viable cells.^41^ Since [^99m^Tc]MIBI is taken up by viable myocardial tissue, but not by necrotic tissue, we expected to see its reduced uptake in the isoproterenol‐treated hearts. However, the acquired SPECT images showed no visible differences. Only with quantitative VOI analysis of the images we could detect a significant reduction in uptake in isoproterenol hearts as compared to the normal hearts. Other groups have reported varying degrees of alterations in [^99m^Tc]MIBI uptake as a result of cardiotoxic agents and myopathies. [^99m^Tc]MIBI uptake has been shown to increase in patients with doxorubicin‐induced cardiotoxicity.^42^ No apparent changes in [^99m^Tc]MIBI uptake were reported between controls and patients with statin‐induced myopathy or congestive heart failure.^43^, ^44^ Because [^99m^]MIBI uptake is dependent on cellular integrity and mitochondrial viability, it is likely that uptake in conditions of cardiomyopathy are dependent on how the injury alters mitochondrial function. The damage caused by isoproterenol treatment is diffuse and widespread in the heart. In the absence of a focal area of damage, we suspect that [^99m^Tc]MIBI is not able to provide differential diagnosis of isoproterenol‐induced myocardial injury.

As a sympathomimetic amine, isoproterenol has potent chronotropic and inotropic effects on the heart. Accordingly, it increases myocardial utilization of oxygen, creating imbalance in supply and demand and resulting in a hypertrophic heart. Isoproterenol‐induced hypertrophy is associated with arrhythmias, myocyte loss, myocardial ischemia, and fibrosis, with progression to heart failure.^45^, ^46^ From the results of this study it appears that isoproterenol‐induced cardiomyopathy impacts cellular metabolism more than perfusion, which results in larger changes in [^18^F]FDG uptake than in [^99m^Tc]MIBI accumulation in cardiac tissue. Although the quantitative uptake values of [^18^F]FDG were significantly altered, the [^18^F]FDG/PET images were more remarkable in showing reduced uptake in isoproterenol‐treated hearts. Previously, Heather et al showed that prolonged dosing of rats with isoproterenol resulted in generalized suppression of myocardial metabolism, including myocardial palmitate oxidation rate, citrate synthase activity, and insulin‐stimulated glycolysis as well as expression of glucose transporter GLUT4.^47^ Glucose uptake in cardiomyocytes is mediated by GLUT1 and GLUT4. ^48^, ^49^ There is evidence to indicate that metabolic remodeling precedes most other pathological alterations and likely plays an essential role in cardiac hypertrophy and heart failure.^50^ At the same time, acute phase of drug‐induced cardiomyopathy may not manifest in considerable changes in myocardial perfusion, unless perfusion deficit is exaggerated by stress.

## 5 CONCLUSION

We demonstrated the utility of [^18^F]FDG/PET to image myocardial damage in a rat model of isoproterenol‐induced injury. To our knowledge, this is the first study which investigated [^18^F]FDG and [^99m^Tc]MIBI uptake in this model of drug‐induced cardiomyopathy. Because of the profound impact of accurate and early detection of MI on patient care, definitive diagnosis of drug‐induced MI is of critical importance. When compared to each other, [^18^F]FDG performed better than [^99m^Tc]MIBI in delineating healthy from damaged myocardium. Although controlled trials will further unravel the role of new PET agents for perfusion imaging ([^18^F]Flurpiridaz and [^18^F]BFPET), it is noteworthy that imaging of myocardial viability, as indicated by intact perfusion, is not always able to predict clinical outcome of revascularization or therapy in MI.

## CONFLICT OF INTEREST

None.

## AUTHOR CONTRIBUTIONS

VA conceived and designed the study; HH, AH, and VA performed the experiments and collected the data; HH and VA conducted the data analysis and wrote the manuscript. All authors have reviewed and approved this manuscript.
